# Filling the Gaps – A Call for Comprehensive Analysis of Extracellular Matrix of the Glial Scar in Region- and Injury-Specific Contexts

**DOI:** 10.3389/fncel.2020.00032

**Published:** 2020-02-20

**Authors:** Jacob Kjell, Magdalena Götz

**Affiliations:** ^1^Division of Physiological Genomics, Biomedical Center, Ludwig Maximilian University of Munich, Munich, Germany; ^2^Institute for Stem Cell Research, Helmholtz Zentrum München, Munich, Germany; ^3^Department of Clinical Neuroscience, Karolinska Institutet, Solna, Sweden; ^4^Departments of Neurology and Neurosurgery, Karolinska University Hospital, Solna, Sweden; ^5^SYNERGY, Excellence Cluster Systems Neurology, University of Munich, Munich, Germany

**Keywords:** brain injury, extracellular matrix, proteomics, glial scar, monocytes, macrophages

## Abstract

Central nervous system (CNS) injury results in chronic scar formation that interferes with function and inhibits repair. Extracellular matrix (ECM) is prominent in the scar and potently regulates cell behavior. However, comprehensive information about the ECM proteome is largely lacking, and region- as well as injury-specific differences are often not taken into account. These aspects are the focus of our perspective on injury and scar formation. To highlight the importance of such comprehensive proteome analysis we include data obtained with novel analysis tools of the ECM composition in the scar and show the contribution of monocytes to the ECM composition after traumatic brain injury (TBI). Monocyte invasion was reduced using the CCR2-/- mouse line and step-wise de-cellularization and proteomics allowed determining monocyte-dependent ECM composition and architecture of the glial scar. We find significant reduction in the ECM proteins Tgm1, Itih (1,2, and 3), and Ftl in the absence of monocyte invasion. We also describe the scar ECM comprising zones with distinctive composition and show a subacute signature upon comparison to proteome obtained at earlier times after TBI. These results are discussed in light of injury-, region- and time-specific regulation of scar formation highlighting the urgent need to differentiate injury conditions and CNS-regions using comprehensive ECM analysis.

## The Glial Scar and ECM

Upon trauma adult mammalian tissue typically scar causing tissue and organ dysfunction. In the central nervous system (CNS) scars affect information processing by several means (Robel and Sontheimer, 2016) including the formation of barriers for re-establishing connectivity (Cregg et al., 2014). Hence scars act as permanent barriers for self-repair and remain an obstacle for therapies enhancing plasticity or neuronal regeneration (Barker et al., 2018).

Scars are typically composed of a cell mixture comprising tissue resident cells, such as different glial cells in the CNS (Adams and Gallo, 2018), and invading cells such as inflammatory cells derived from the immune system, e.g., monocytes (Orr and Gensel, 2018). In addition, in some injury paradigms and CNS regions (e.g., after spinal cord injury) fibroblasts and/or pericytes accumulate in the core of the injury site (Göritz et al., 2011; Soderblom et al., 2013). Notably, this cell mixture differs profoundly depending on the injury type (TBI, stroke, amyloid deposition, autoimmune-reaction) and the CNS region. For example, in spinal cord injury fibroblast-like cells settle in the lesion core that is shielded/surrounded by reactive glial cells, such as astrocytes (Kjell and Olson, 2016). This is can be very different in stab wound brain injury with prominent reactive gliosis and little to no detectable fibrosis (Frik et al., 2018). This is also the case, when the brain injury reaches into the White Matter (WM) (Mattugini et al., 2018) that is often less affected in brain injury as it is buried deep in the brain below the Gray Matter (GM). After spinal cord injury WM is always affected first as it is located at the surface. Effects on WM are thus one of the many major differences upon injury inflicted to these very distinct regions. Notably, monocyte invasion continues into much later stages after the injury when WM is affected, while newly invading monocytes can no longer be detected 5–7 days after injury of GM only (Mattugini et al., 2018.). Thus, the cellular composition of the wound and scar differs profoundly in a region-specific manner in the CNS.

Given that all of these different cells communicate and interact by a plethora of cell surface signaling pathways and secretion of specific proteins it is essential to unravel this complex proteome, not the least to also understand how injury- and region-specific extracellular matrix (ECM) composition contributes to scar formation. A suitable approach is to deplete one population and then examine relevant changes. For invading monocytes, this can be done by blocking or deleting the CCR2 – a receptor absolutely essential for invasion of monocytes into the brain (Saederup et al., 2010). Intriguingly, lack of monocyte invasion shows profoundly different outcomes in different injury conditions and distinct CNS regions. Preventing monocyte invasion after ischemic stroke has been reported to worsen the hemorrhagic consequences and reduce the long-term recovery (Gliem et al., 2012; Wattananit et al., 2016). However, in TBI models the prevention of monocyte invasion has reduced the volume of the injury-affected region and improved cognitive function (Hsieh et al., 2014; Morganti et al., 2015). These data may well reflect, the differences in ECM composition in different regions and injury conditions.

Extracellular matrix changes have mostly been examined after spinal cord injury aiming to understand how they affect the restoration of ascending and descending axonal connections (Didangelos et al., 2016; Bradbury and Burnside, 2019). Several ECM components have been found to be inhibitory for repair, especially chondroitin sulfate glycosaminoglycan-chains (GAG-chains) on proteoglycans at the injury site. These sugar-chains have proven inhibitory to axon growth and digesting them enzymatically or keeping them from stable growth cone interactions improves regeneration (Bradbury et al., 2002; Bartus et al., 2014; Lang et al., 2015). The Tenascin glycoproteins have been found to be upregulated in the same region as the CSPG, where Tenascin-R is a component normally part of the Perineuronal nets (PNN) (Deepa et al., 2006; Carulli et al., 2010) and Tenacin-C (Tn-C) is an inflammation-associated ECM protein upregulated following CNS injury (Roll and Faissner, 2019). Other commonly reported scar components are those of the fibrotic scar that are of similar composition to the basal membrane, containing e.g., collagen, laminins, and fibronectin. Moreover, injury is associated with the degradation of such core ECM proteins (Roll and Faissner, 2014). Responsible for this are peptidases, include the elastases and matrix metallopeptidases. In spinal cord, another catalytic ECM-associated protein group called cathepsins has recently been highlighted using proteomics and transcriptomics as potential ECM regulators (Tica et al., 2018), providing an example of the multitude of unexplored avenues for ECM and its associated proteins in CNS injury.

## Monocyte-Dependent ECM in the Glial Scar

**FIGURE 1:**
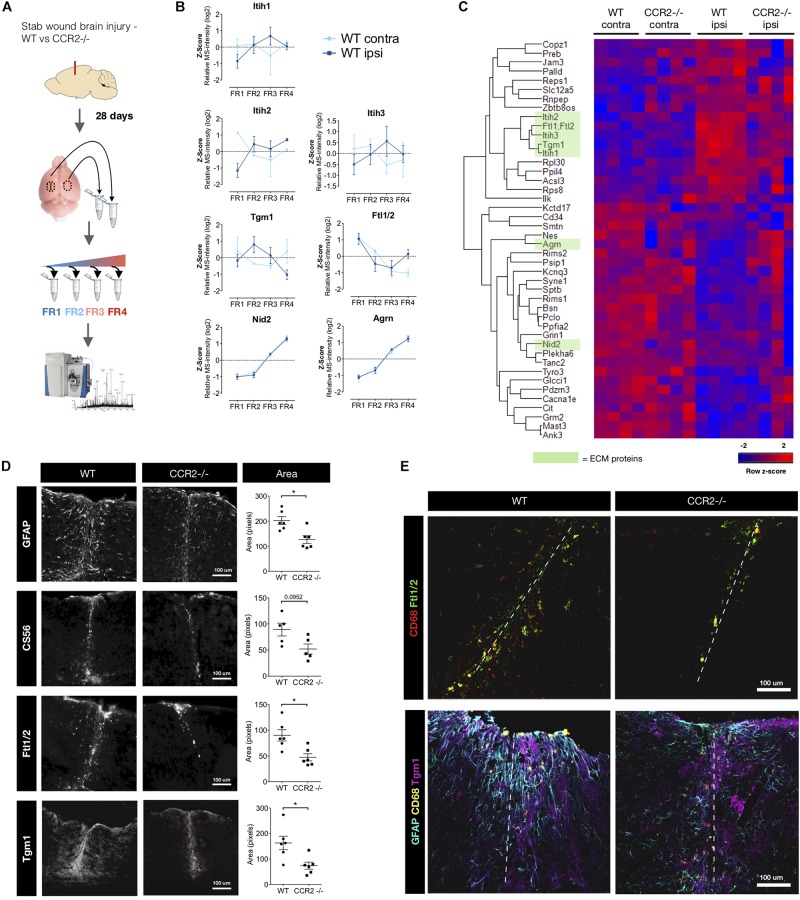
Macrophages contribute to the ECM-component of the scar after stab wound injury. **(A)** Schematic of the proteome comparison 28 days after brain stab wound injury in wild-type mice compared to CCR2-/- mice using step-wise detergent-decellularization protocol, named quantitative detergent solubility profiling (QDSP). Mouse brain picture courtesy: National Science Foundation. **(B)** The QDSP method subjects the tissues to increasing strength of detergent lysis and results in four individual fractions. Fraction measurements have are here compared for ECM and secreted proteins in the contralateral and the ipsilateral side of the injured wild-type brains, which returned to more normal levels in the CCR2-/- mouse. Overall the solubility changes trend toward a more insoluble nature, although not exclusively insoluble. Notably, while the total abundance of Nid2 and Agrn normalized (got more abundant) in the CCR2 -/- mice, there was no difference in their solubility profiles. **(C)** Combined fraction analysis reveal that the overall protein changes are normalized in the CCR2 -/- mice compared to wild-type mice (*n* = 4 per group). Heatmap displays proteins that had similar abundance in the contralateral samples (*t*-test, *p* ≥ 0.05), while being significantly different when comparing contralateral to ipsilateral side of the injured wild-type mouse brain (*t*-test, *p* ≤ 0.01). ECM and secreted proteins are highlighted in green. **(D)** Quantifications of the immunoreactive tissue-area at the injury site confirm that Ftl1/2 and Tgm1 are reduced in the CCR2-/- mice closer to levels contralateral to the injury. This is in line with the reduced spread of other glial scar markers such as GFAP and CS56. * *p* ≤ 0.05. **(E)** Ftl1/2 primarily co-localizes with the macrophage marker CD68, while Tgm1 instead is predominantly found in the area of reactive astrocytes (GFAP + cells).

Inflammation is part of the cascade of events that follows traumatic injury and has also been found to regulate the extent of the scarring after TBI in cerebral cortex GM (Frik et al., 2018). Of the multiple invading immune cells, monocyte comprises the largest group, and surprisingly little is known about the role of macrophages in affecting the ECM composition at the scar. With scarring reduced after stab wound injury in the CCR2-/- cerebral cortex (Frik et al., 2018) we investigated which ECM components may be affected by the virtual absence of macrophages in the brain parenchyma after injury in this mouse line. We used state-of the-art proteomics (Cox and Mann, 2008; Mann et al., 2013; Cox et al., 2014; Kulak et al., 2014; Tyanova et al., 2016) and the quantitative detergent solubility profile (QDSP) method, in order to also investigate the architecture and composition of the ECM. The QDSP method analyses all the tissue lysates fractions after the tissue has been sequentially processed in increasing strength of detergent (see Supplementary Material or Kjell et al., 2020), with the most detergent-insoluble proteins in a separate fraction (fraction 4 – see Figure 1A). Basically, it is a tissue decellularization that also provides information regarding the intermediates solubilities as proteins are identified and quantified in all (four) fractions using label-free mass spectrometry. The ECM proteins are identified in the data-set by the currently most comprehensive annotation for the ECM proteins – the matrisome annotation that uses a combination of proteomic measurements from decellularized tissue and *in silico* prediction to identify the ECM proteins (Naba et al., 2012). The advantage of analyzing all detergent-fractions in the QDSP method is that it allows determining how the total abundance of a protein is distributed in the different fractions and how this solubility profile shifts under different conditions. This provides crucial information regarding changes in ECM protein distribution, e.g., from basal lamina (highly insoluble) to interstitial space (soluble) after trauma. Indeed, Transglutaminase 1 (Tgm1) changes its solubility profile reducing the insoluble fraction after injury with a peak in fraction 2, i.e., becoming more soluble. Conversely, the inter-alpha-trypsin inhibitors 1,2,3 (Itih1,2,3) became rather less soluble at the scar stage (28 days post injury; dpi) after TBI (Figure 1B).

In our investigation of the adult neurogenic niche (Kjell et al., 2020), also using the QDSP protocol, we found that ECM proteins, such as Tn-C, are more soluble compared to the brain parenchyma. Tn-C is also highly soluble in the scar region analyzed by QDSP at 28 dpi here, which is opposite to its insoluble nature in lung injury and atherosclerotic plaques (Schiller et al., 2015; Wierer et al., 2018). These are examples of how any quantification and information regarding ECM architecture would be lost without adopting a protocol that allows composition-dependent sample analysis.

Next, we examined combined fraction analysis to determine total abundance comparisons for any protein (Figure 1C). To identify proteins regulated by invading monocytes and their influence in scar formation, we compared proteins of similar magnitude in the non-injured contralateral side of the WT and CCR2-/- mice brains (two-tailed *t*-test, *p* ≥ 0.05) that were significantly changed following injury in the WT mice 28 days after stab wound injury (two-tailed *t*-test, *p* ≤ 0.01) (Figure 1C). We found four ECM proteins and one secreted protein that were elevated with injury, but reduced in the CCR2-/- mice (green bar in Figure 1C). These were the above mentioned Tgm1, Itih1,2,3 and the ferritin light chain proteins 1 or 2 (here referred to as Ftl1/2). Notably, all of these have previously received little if any attention with regard to brain injury and glial scarring. Tgm1 is part of the cross-linking enzyme family of transglutaminases that have many roles, including the regulation of tissue stiffness when crosslinking ECM (Gundemir et al., 2012; Majkut et al., 2013). Immunostaining for Tgm1 was found to be rather diffuse around the injury in the entire area dominated by astrogliosis (Figures 1D,E). Taken together, this may suggest that the multi-function enzyme Tgm1 crosslinks cell surface proteins and/or less detergent resistant ECM proteins and/or prevails in the cytoplasm where it has intracellular functions (Eckert et al., 2014).

Itih 1,2,3 are hyaluronic acid binding proteins that can act as protease inhibitor and are often present with inflammation. Ftl1/2 binds ferric ions that would otherwise be toxic to the cells. These proteins could originate from the blood (Geyer et al., 2016). However, bioinformatic comparison from our previous publications with proteomes from the stab wound at 3 and 5 days after injury, hinted that this was unlikely (Frik et al., 2018; Mattugini et al., 2018). In these proteomes, blood proteins are highly abundant at 3 days after stab wound injury, but are instead reduced at 5 days. At 5 days, we find Tgm1, Itih3, and Ftl1/2 to be more abundant, while the overall blood-related proteins have decreased. Furthermore, this highlights that much of the ECM changes remain from, or have similar composition to, a subacute stage after injury.

We confirm the presence of Ftl1/2 and Tgm1 in the tissue with immunohistochemistry. Our area-coverage analysis suggests the reduced abundance of these proteins in the CCR2-/- mouse stab wound injury site at 28 days is due to being restricted to a smaller area (Figure 1D). Most of the Ftl1/2 could be attributed to CD68+ macrophages or activated microglia and was present in a similar area to the chondroitin sulfate GAG-chains at the injury site (Figures 1D,E). Given that Ftl1/2 is secreted and became more insoluble at the scar (Figure 1B), we propose this protein to be a matrisome-associated protein. An interesting possibility is that it may be bound to the ECM of the vasculature to capture Fe ions prior to entering the brain parenchyma to prevent the induction of toxic phospholipid oxidation products that lead to ferroptosis of cells at the injury site (Stockwell et al., 2017; Conrad and Pratt, 2019). Neurons – also in direct reprogramming from glial cells – can be particularly vulnerable to ferroptosis as recently shown (Gascón et al., 2016).

Interestingly, the block of proteins that are reduced ipsilateral compared to contralateral by injury are often associated to synapses (Figure 1C, e.g., Agrin, Cacna1e, Kcnq3, Mast3) indicative of the synapse loss persisting in the scar region of the injury site. Intriguingly, this loss is alleviated in the injury site of the CCR2-/- mice consistent with the notion that the scar is indeed reduced and the absence of monocyte invasion is beneficial for neuronal network recovery (Dimitrijevic et al., 2007; Hsieh et al., 2014; Morganti et al., 2015; Frik et al., 2018). We also see changes in the solubility of synaptic proteins (e.g., Gria2-3, Olfm3, Glgap1, and Vamp1), while we did not see an obvious change in the solubility of PNN proteins in the scar stage between the genotypes. Our previous QDSP analysis of PNN proteins in the uninjured cerebral cortex had revealed that they are typically not insoluble (in fraction 4), but rather belong to a less detergent resistant category (fractions 2–3; Kjell et al., 2020). Taken together, lack of monocyte invasion affects ECM proteins in a long-term manner toward a state closer to the un-injured contralateral site, thus normalizing the scar ECM.

## ECM Origin and Scarring Zones

**FIGURE 2:**
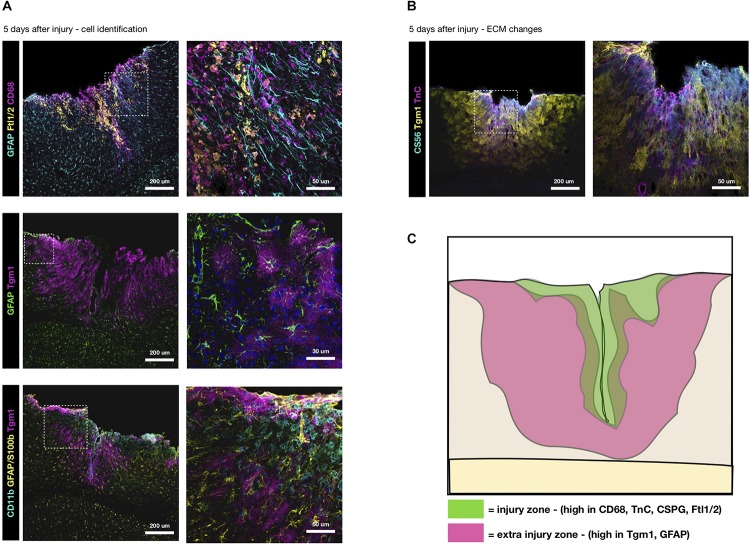
Distinctive ECM zones at 5 days after TBI. **(A)** Tgm1 and Ftl1/2 immunsotaining at 5 dpi, the peak of their deposition (Frik et al., 2018). Ftl1/2 is mostly found around CD68+ macrophages or activated microglia at the center of the injury site. Instead Tgm1 is present in a wider area overlapping with the region of GFAP+ reactive astrocytes, suggesting astrocytes may be a likely candidate for much of the Tgm1 expression/deposition. Interestingly, Tgm1 seems to surround the macrophage/activated microglia dense area suggestive of regionalization. **(B)** Tn-C and CS56, two ECM markers typical for the reactive glia, are localized to the center of the forming scar, while Tgm1 is more peripheral to it. **(C)** Here, in a conceptual summary of the subacute wound after stab wound injury of the brain, we suggest there are two primary zones (at the injury site and peripheral to the injury site) with different changes in the extracellular microenvironment that are fundamental to the final composition of the glial scar.

Comparing previous proteomes obtained at the acute stages 3 and 5 dpi (Frik et al., 2018; Mattugini et al., 2018), we find scar-related ECM proteins peak at 5 days. At this time, we find Ftl1/2 to be present on and around CD68+ macrophages, although it seems to only be a subset that is responsible for the Ftl1/2 secretion (Figure 2A). Hence, we suggest that reduced levels of Ftl1/2 are a direct consequence of preventing the invasion of monocytes. Changes in Tgm1 on the other hand, rather seem to be an indirect consequence, since Tgm1 is not produced by macrophages, but rather astrocytes, as seen by immunostaining (Figures 2A,B). Indeed, Tgm1 has been proposed as a marker for neuroprotective astrocytes (Liddelow et al., 2017). Interestingly, Tgm1 spreads much further than the area densely populated with macrophages/activated microglia, giving credit to the idea that Tgm1 would be part of a neuroprotective response, possibly by maintaining the tissue integrity. The tissue of the glial scar is softer at the area affected by macrophages (Moeendarbary et al., 2017) and perhaps tissue more peripheral to the injury would also succumb to a similarly soft mechanical signature if Tgm1 was not present to counteract this by crosslinking ECM proteins.

Comparing the localization of Tgm1 with Tn-C and CSPG at 5 dpi, we find the increased levels of Tn-C and chondroitin sulfate GAG-chains (CS56) to be more associated to the core area of the injury with dense macrophage infiltrates, rather than the astrogliosis (Figure 2B). Hence, our results suggest that there are zones with different ECM composition in the scar-forming region (Figure 2C). The ECM and cell markers also suggest these zones may represent neurotoxic (core) and neuroprotective (surround) areas. Clearly, invasion of monocytes increases the neurotoxic core of the forming scar and its specific ECM, both directly and indirectly.

## Complexity in Investing the Glial Scar

In any organ, scarring is a process that renders the respective part of an organ chronically non-functional. Scars are typically different from normal tissue in their ECM composition and hence a lot can be read from the changes in ECM in different tissue under abnormal conditions. However, in most cases only few ECM proteins are monitored as “representative” of scar formation and a comprehensive analysis is missing. Proteomics now offer a robust way to detect abundances of a large set of ECM, even in small tissue samples with maintained depth of detection and identification. Here we combined the proteomics with a protocol that gives a good indication on the cellular compartment of all proteins. However, there may be further aspects of the ECM to be resolved with other sample-separation protocols, including investigations concerning the sugar-chain composition with glycomics or glycoproteomics. Comparative proteome-to-transcriptome analysis may yield further insights (Schiller et al., 2015; Angelidis et al., 2019; Kjell et al., 2020), specifically with single-cell RNA sequencing data that may allow identification of the cellular source of specific ECM proteins. For example, our analysis of the adult neurogenic niche shows that quiescent NSCs are by large the main contributor to the ECM composition of this niche (Kjell et al., 2020).

Moreover, proteome analysis at different times after stab wound injury unraveled that scar formation is determined at a subacute stages. When we compared the proteome of the injury site at 5 and 28 dpi between WT and CCR2-/- mice, we observed that many scar-related factors (such as enzymes for gycosaminoglycanes, but also many scar-resident proteins, such as Ftl1/2, Itih) are already present at 5 dpi and higher in WT. At this time the injury site and reactive gliosis region surrounding the injury is not yet obviously different between the genotypes, but the proteomic composition including ECM proteins is already profoundly changed in the absence of monocyte invasion (Frik et al., 2018).

Given the potent effects of monocyte invasion on scar formation, it is important to note the differences in monocyte invasion in GM and WM regions. This can best be demonstrated when GM and WM injury are directly compared in the same brain region, as was recently done for the cerebral cortex using the GM injury paradigm described above and extending it into the WM (Mattugini et al., 2018). This revealed a much prolonged and bi-phasic monocyte invasion up to 2–3 weeks after injury in the WM, very much reminiscent of data obtained after spinal cord injury (Blight, 1992; Popovich et al., 1997; Beck et al., 2010). As WM sits at the surface of the spinal cord, it is always affected upon mechanical injury in this region, while brain injury is often restricted to GM given its location at the brain surface. Along with different patterns of monocyte invasion, many other aspects of gliosis, such as NG2 glia proliferation, were also different in brain TBI comprising the WM compared to GM only injury conditions (Mattugini et al., 2018). These data highlight that results obtained in one CNS region, such as the spinal cord, can not simply be extended to other regions, such as the cerebral cortex. Likewise, results obtained in one injury paradigm can not simply be extended to others as highlighted by the diversity of effects obtained by deletion or blocking of CCR2 in different injury paradigms. Unfortunately, this obvious message is all too often ignored.

These profound region- and injury-specific differences can also be observed when considering the zonation at the subacute injury site and the aspects of it that persist as part of the scar. Here we described two partly overlapping zones consisting of different cell types and ECM proteins in an injury largely lacking fibrosis. For other injury types with a fibrotic core it will be important to understand the different ECM composition of the fibrotic ECM and the surrounding gliotic one. This could also be done by using proteomic techniques analyzing the proteins directly from tissue sections such as MALDI-TOF (Lahiri et al., 2016; Quanico et al., 2018). Although fresh frozen tissue is preferred for proteomics, analyzing fixed tissue is now feasible, while maintaining a reasonable dept (Coscia et al., 2018) allowing exploration of ECM in patient samples. For example, such investigations have recently elucidated the role of the ECM-rich stromal compartments for cancer progression (Eckert et al., 2019). In addition to extending to human samples, it will be important to extend ECM analysis to samples of vertebrates with scar-less wound healing also after brain injury, such as the zebrafish (Baumgart et al., 2012; Kizil et al., 2015). Such data could teach us the composition of an extracellular environment that mediates wound healing without scar formation and allows neurogenesis and the integration of the new neurons. Characterizing such ECM changes may then help to steer ECM composition in mammalian brains toward scar-less wound healing supporting also neuronal replacement therapies.

## Data Availability Statement

The raw data supporting the conclusions of this article are available via ProteomeXchange (Vizcaino et al., 2013) with identifier PXD017478.

## Ethics Statement

The animal study was reviewed and approved by the Government of Upper Bavaria (Regierung von Oberbayern).

## Author Contributions

MG and JK wrote the perspective. MG conceived the project. JK and MG conceptualized and planned the project. JK performed all experiments and analysis.

## Conflict of Interest

The authors declare that the research was conducted in the absence of any commercial or financial relationships that could be construed as a potential conflict of interest.
