# Supplementary material for: Filling the Gaps – A Call for Comprehensive Analysis of Extracellular Matrix of the Glial Scar in Region- and Injury-Specific Contexts
Source: Front Cell Neurosci. 2020 Feb 20;14:32. doi: 10.3389/fncel.2020.00032 (PMC7050652; doi:10.3389/fncel.2020.00032)
Supplement: Supplementary file 1 [file Data_Sheet_1.pdf]

Supplementary material information

Animals

All experimental procedures were performed in accordance with German and European Union guidelines and were approved by the government of upper Bavaria (Regierung von Oberbayern, #ROB-55.2-2532.Vet_02-16-210). For the proteomics experiments, only male C57Bl6/J mice between 8-10 weeks were used. For IHC, both male and female C57Bl6/J mice between 8-12 weeks were used. Mice were fed ad libitum and housed with a 12/12 h light and dark cycle.

Surgery

Stab wound brain injury was performed as described in Frik et al., 2018. In brief, mice were anasthetised and the head was secured in a stereotactic apparatus before being subjected to a parasagittal stab wound injury (0.6 mm deep and 1.5 mm long) on the right cortical hemisphere using an ophthalmological knife. Animals were sacrificed at 28 days after injury for proteomics and 28 and 5 days after injury for immunohistochemistry.

Immunohistochemistry

Brains were extracted after mice were anaesthetized by intraperitoneal injection of ketamine (100 mg per kg of body weight) and xylazine (10 mg per kg of body weight) and then transcardially perfused first with PBS followed by 4% Paraformaldehyde (PFA). Brains were post-fixed in 4% PFA for 16h at 4°C and then put into 30% sucrose solution for 2 days. The fixed brains were cut at 20 µm at cryostat (Leica CM3050S). Sections were incubated in primary antibodies in PBS containing 0.4% Triton X-100 and 10% normal goat serum (NGS) overnight at 4°C, washed and incubated with secondary antibodies in PBS containing 10% NGS for 2 hours at RT. Astrocytes were visualized with GFAP (ms, 1/500, Sigma, #63893) and S100b (ms, 1/500, Sigma, L1516-2MG), Macrophages/activated microglia with CD68 (rat, 1/200, Abcam, ab5344) and Cd11b (rat, 1/200, AbD Serotec, MCA74G). Other primary antibodies were Tgm1 (rbt, 1/3000, Thermofisher, PA5-59088), Ftl1 (rbt, 1/500, Abcam, ab69090), CS56 (ms, 1/100, Abcam, ab11570), and Tn-C (rat, 1/100, Thermofisher, MA1-26778). Secondaries were of complementary species to the primary antibodies and alexafluor 488, 555, and 647 (conc. 1/1000, Thermofisher, A-11001). Fiji (fiji.sc) was used to determine area coverage of immunoreactivity 28 days after injury in the ipsilateral side of WT and CCR2-/- mice brains (n=6, each n is an average from 3 representative sections per brain). Photomicrographs for quantification were acquired using fluorescent microscope AXIO Imager

M2m (Zeiss) and evidence for colocalization was acquired using confocal microscope LSM 710 (Zeiss).

Proteome measurments and analysis

The QDSP sample preparation was performed as in Schiller et al. 2015. In brief, mice were sacrificed by cervical dislocation (n=4 per mouse type) and brains were subsequently extracted and the injury site and corresponding area on the contralateral side was removed using a 2.5 mm biopsy punch. The WM was removed and each sample had 0.5 mm removed from each side parallel to the stab wound. Samples were homogenized using in PBS (with protease inhibitor cocktail and Ethylenediaminetetraacetic acid (EDTA)) and stored in -80 °C until further tissue processing. When thawed, samples were centrifugated and the supernatant (protein fraction 0) was collected. We sequentially resuspended the pellet in three detergent buffers, each followed by centrifugation for 20 min at 16,000 g. The samples were incubated in buffer 1 (150 mM NaCl, 50 mM Tris–HCl (pH 7.5), 5% glycerol, 1% IGEPAL, 1 mM $MgCl_2$, protease inhibitors (+EDTA), 1% benzonase, 1× phosphatase inhibitors) and buffer 2 (150 mM NaCl, 50 mM Tris–HCl (pH 7.5), 5% glycerol, 1.0% IGEPAL, 0.5% sodium deoxycholate, 0.1% SDS, 1× protease inhibitors (+EDTA), and 1% benzonase) for 20 min on ice, and in buffer 3 (500 mM NaCl, 50 mM Tris–HCl (pH 7.5), 5% glycerol, 1.0% IGEPAL, 2% sodium deoxycholate, 1% SDS, 1× protease inhibitors (+EDTA), and 1% benzonase) for 20 min at RT. The supernatant from the buffer treatment resulted in fractions 1, 2, and 3, and residual insoluble material resulted in fraction 4. Fraction 0 and 1 were combined and from here on labelled as fraction 1. All four fractions were precipitated (80% acetone) and sonicated (5x30 sec) (Bioruptor, model UCD-200, Diagenode). Then followed combined alkylation/reduction (100 mM Tris-HCl (pH 8.5), 6M GDmCl, 10 mM TCEP, and 50 mM 2-chloroacetamide) for 15 min at 99 °C. After sonication (10 x 30 sec) we determined protein concentration (Micro BCA protein assay kit, ThermoFisher Scientific). Enzymatic digestion was done at 37 °C for 2h with LysC (1/50) and then with LysC (1/50) and Trypsin (1/25) overnight. Samples were acidified (TFA to 1% of sample) and then desalted using the StageTip method (Kulak et al. 2014) with SDB-RPS filters. Peptides were eluted in 5% ammonia (from 25% stock solution) and 80% acetonitrile followed by liquid evaporation using speed-vac.

Mass spectromtery

2 µg of peptides were loaded in buffer A (0.1% (v/v) formic acid). Peptides were separated by a 2h gradient in a 50 cm long C18 column (75 µm inner diameter filled in house with ReproSil-

Pur C18-AQ 1.9-lm resin (Dr. Maish GmbH)) and eluted in 5–60% buffer B (0.1% (v/v) formic acid, 80% (v/v) acetonitrile) at a flow rate of 250 nl/ min using a nanoflow UHPLC (Easy nLC, Thermo Fisher Scientific) online coupled to the mass spectrometer (Q Exactive HF Orbitrap, Thermo Fisher Scientific). Each sample was followed by a wash with buffer B and recalibration with buffer A. Survey scans had a resolution of 70,000 at m/z 400 with a maximum injection time of 20 ms. Target value for the full scan MS spectra was 3×10^6 and isolation window of 1.6 m/z and the 10 most abundant precursor ions were chosen for fragmentation. MS/MS scans had a resolution of 17,500 at m/z 400 with a maximum injection time of 120 ms. Ion target value for the MS/MS scan was 1×10^5.

The mass spectra was processed using MaxQuant (coxdocs.org/doku.php) (Cox & Mann 2008). The spectra were searched against the mouse Uniprot sequence database (uniprot.org) using the Andromeda search engine. Cysteine carbamidomethylation was set as fixed modification while variable modifications included hydroxylation of proline and methionine oxidation. For both protein and peptide level we set FDR to 0.01 and only peptides with an amino-acid length of seven or more were considered. We used matching between runs, but restricted quantification to a minimum of two unique peptides. We quantified proteins with the Label-Free Quantification (LFQ) (Cox et al. 2014), allowing quantification with peptide or protein fractionation while maintaining high accuracy (Cox et al. 2014). Proteins were considered valid with two peptide ratios.

Bioinformatic analysis and statistics

Graphpad Prism (version 5) was used for visualization of the profiles generated with the QDSP method (WT mice ipsilateral vs contralateral). Bioinformatic analysis was performed using the Perseus software (coxdocs.org/doku.php) (Tyanova et al. 2016). For the combined fraction analysis we filtered the data for at least 6 valid values in total prior intensity imputation. Imputation of missing values for the protein fractions of each region was done by random selection according to a normal distribution with negative shift of 1.8 standard deviations from the mean and with a width of 0.4 standard deviations. The data in the heatmap has gone through hierarchical cluster analysis (rows) and consist of the proteins similar in WT and CCR2-/- contralateral sides (t-test, $p \geq 0.05$), while being significantly altered comparing ipsilateral and contralateral side of the WT mice (t-test, $p \leq 0.01$).

Data availability

The mass spectrometry proteomics data have been deposited to the ProteomeXchange Consortium (http://proteomecentral.proteomexchange.org) via the PRIDE partner repository (Vizcaino et al., 2013) with the dataset identifier <PXD017478>. The analyzed QDSP proteome data presented in this Perspective can be made available upon request.
